# Development of a Lateral Longitudinal Arch Evaluation Method for the Foot Using Ultrasonography: Validation With Radiography and Verification of Intrarater and Interrater Reliability

**DOI:** 10.1002/jfa2.70039

**Published:** 2025-02-25

**Authors:** Daichi Kawamura, Takashi Komatsu, Masanobu Suto, Hikaru Narita, Yasuyuki Umezaki, Saki Takahashi, Hiroshi Shinohara

**Affiliations:** ^1^ Graduate School Aomori University of Health and Welfare Aomori Japan; ^2^ Shobukai Medical Corporation Komatsu Orthopedic and Sports Clinic Aomori Japan

**Keywords:** cuboid height, lateral longitudinal arch, radiography, ultrasonography

## Abstract

**Introduction:**

The lateral longitudinal arch (LLA) is an essential structure of the foot. However, LLA evaluation methods remain underexplored compared to those of the medial longitudinal arch (MLA). This study sought to develop a method for measuring the cuboid height, the keystone of the LLA, using ultrasonography and to verify its correlation with radiography, as well as intrarater and interrater reliability.

**Methods:**

This cross‐sectional study included 21 university students (14 males and seven females). The cuboid height was measured using radiography and ultrasonography. The validity of ultrasonographic measurements was assessed through correlation with radiographic measurements and Bland–Altman analysis. Intrarater and interrater reliabilities were evaluated using intraclass correlation coefficients (ICCs).

**Results:**

A strong correlation was observed between cuboid heights measured using radiography and ultrasonography (*r* = 0.98, *p* < 0.01). The Bland–Altman analysis revealed a fixed bias of −0.71 mm (95% confidence interval [95% CI]: −0.96 to −0.46 mm). Intrarater and interrater reliability for ultrasonographic measurements were almost perfect, with ICCs of 0.98 and 0.99, respectively.

**Conclusions:**

Cuboid height measurements using ultrasonography demonstrated high validity and reliability. This method offers a noninvasive and cost‐effective alternative to radiography, with potential clinical applications in the evaluation of LLA and related conditions such as cuboid syndrome and lateral foot injuries.

## 1 Introduction

The human foot is composed of 26 bones and forms three types of arched structures: the medial longitudinal arch (MLA), the transverse arch, and the lateral longitudinal arch (LLA) [1, 2]. Among these, the MLA has been widely studied for its role in interventions using insoles, basic research concerning the windlass mechanism, and its relationship with sports activities, such as walking and running [3, 4, 5, 6, 7, 8]. Several methods have been developed for evaluating the MLA, including shape‐based assessments and simpler techniques using electronic calipers to measure parameters such as navicular height and dorsum height, which are commonly used in clinical and research settings [9].

In contrast, research surrounding the LLA has been limited to a small number of studies, including evaluations using radiography and dynamic analyses during jump landings using cineangiography [10, 11]. Despite its anatomical importance, the functionality and clinically applicable evaluation methods for the LLA remain underexplored, making it one of the less‐studied structures of the human body.

Sports disorders related to the cuboid bone and LLA include cuboid bone syndrome and fifth metatarsal fatigue fractures, and the evaluation of the cuboid bone and lateral longitudinal arch for these disorders has been done subjectively by the examiner or manually [12, 13, 14]. Risk factors for cuboid syndrome include the pronated foot [14], and for the fifth metatarsal stress fracture, the high arch and calcaneal inversion and eversion have been cited as risk factors [15]. Thus, foot morphology has a high potential for causing sports injuries of the fifth column of the foot, and evaluation of static foot morphology may contribute to the prevention of these injuries. However, as mentioned above, subjective and manual methods of evaluating the LLA and cuboid height have been commonly used, and quantitative evaluation has not been performed.

Recently, significant progress has been made in developing methods to evaluate the morphology and structure of foot and ankle muscles using ultrasonography [16, 17]. Notably, Shinohara et al. successfully measured the MLA using ultrasonography, demonstrating high validity compared to measurements obtained using radiography [17]. Unlike radiography, ultrasonography offers advantages such as cost‐effectiveness, absence of radiation exposure, and the ability to safely measure musculoskeletal structures. In recent years, a portable US has also been developed, which enables immediate evaluation of athletic equipment at nonmedical facilities, such as sports sites where large equipment such as X‐ray diagnostic imaging equipment, computed tomography (CT), and magnetic resonance imaging (MRI) are not permanently available, and also enables measurement of a large number of athletes in a short period of time. The system will enable immediate evaluation of athletic equipment and measurement of a large number of athletes in a short period of time. Unlike these devices, US is one of the devices that can be used by physical therapists, and it is possible to describe deep tissues that are difficult to palpate and to confirm the effects before and after rehabilitation and training.

In this context, this study aimed to measure the cuboid height using ultrasonography and to verify its correlation with radiography, as well as intrarater and interrater reliability of the measurements. The ability to quantitatively measure the cuboid bone in this study is expected to facilitate the evaluation of the lateral aspect of the foot, specifically the fifth ray. Measurement of cuboid height in US has clinical significance in that it can be used as an indicator for insoles that supplement cuboid height for the foot and for determining the effectiveness of rehabilitation for the muscle groups that contribute to the fifth column of the foot. In terms of research development, the ability to evaluate the fifth column of the foot is expected to lead to the elucidation of foot biomechanics and the development of sports injuries caused by the fifth column of the foot.

## 2 Methods

### 2.1 Participants

This cross‐sectional study was approved by the ethics committee of the Komatsu Orthopedic Sports Clinic (Ethics Approval Number: 2024.02). Twenty‐one university students (14 males, 7 females; mean age 21.95 ± 0.90 years; height 167.10 ± 9.00 cm; weight 61.57 ± 13.10 kg; foot length 24.46 ± 1.51 cm) participated in the study. The exclusion criterion was the presence of an acute orthopedic condition.

### 2.2 Procedures

Measurements were conducted over 3 days. On the first day, measurements were taken using radiography. On the second day, a single fourth‐year physical therapist (D.K.) performed measurements using ultrasonography. On the third day, two fourth‐year physical therapists (D.K. and Y.U.) performed ultrasonography measurements.

### 2.3 Radiography

**FIGURE 1:**
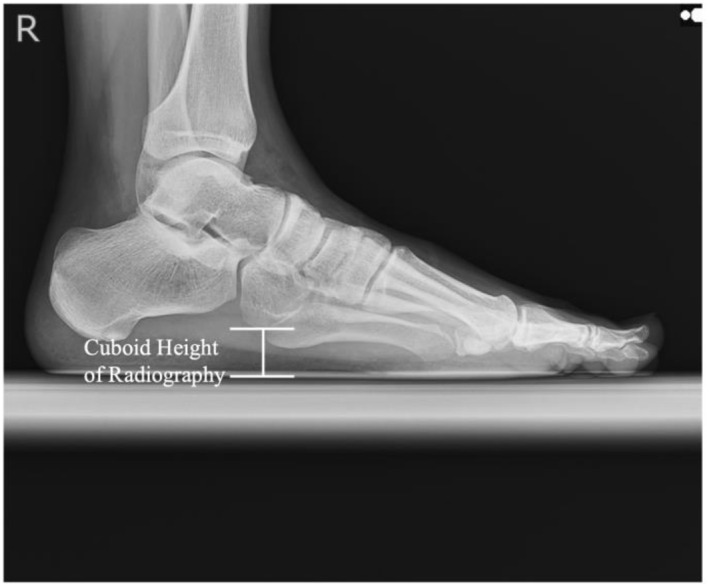
Calculation of the cuboid height using radiography.

Following the procedures described by Gwani, Asari, and Mohd Ismail, the lateral aspect of the right foot of all participants was imaged using radiography [11]. Participants stood on a specially designed platform, 20‐cm high, with a 15‐cm gap between their feet, ensuring that both feet were parallel and evenly loaded. A radiography cassette film was placed parallel to the medial border of the right foot and perpendicular to the ground. The X‐ray tube was positioned 100 cm from the cassette and centered horizontally on the lateral malleolus. The exposure settings were standardized to 6.3 mAs and 55 kV for all participants. The captured images were analyzed using INFINITT PACS (INFINITT Japan, Japan) to calculate the cuboid height, which is defined as the distance from the top of the platform to the lowest point of the cuboid (Figure 1). To minimize measurement errors, the cuboid height was calculated three times from the same image, and the mean value was used as the representative value.

### 2.4 Ultrasonography

**FIGURE 2:**
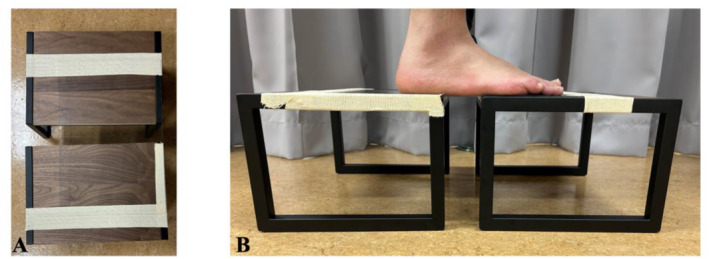
Custom standing platform used for ultrasonographic measurements. (A) PVC plate bridging two 20‐cm‐high platforms with a 6‐cm gap. (B) Side view of the platform setup showing the probe inserted through the gap during the measurement.

Ultrasonographic measurements were performed using an ultrasound imaging device (SONIMAGE HS‐1, KONICA MINOLTA, Japan) with a 10‐MHz linear probe (L18‐4, KONICA MINOLTA, Japan). The cuboid bone of the right foot was imaged from the plantar aspect under the same conditions as those used for radiography. Consistent pressure and angle applications during probe placement are crucial to avoid measurement errors caused by variations as small as 2° [18, 19]. A custom‐built measuring platform was constructed based on previous studies [20]. The platform consisted of two 20‐cm‐high standing platforms with a 6‐cm gap between them, bridged by a 1‐mm‐thick polyvinyl chloride (PVC) plate (Figure 2). The participants stood evenly loaded on the two platforms, ensuring the fifth metatarsal tuberosity and cuboid bone were positioned over the 6‐cm gap. The feet were spaced 15 cm apart and kept parallel, consistent with radiographic conditions. Ultrasound gel was applied between the foot and PVC plate, as well as between the plate and probe.

**FIGURE 3:**
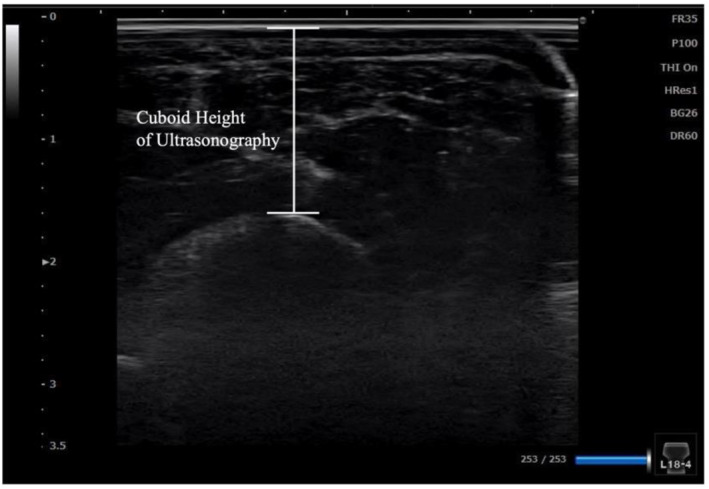
Calculation of the cuboid height using ultrasonography.

Cuboid measurements began with imaging of the fifth metatarsal tuberosity, followed by proximal and medial adjustments of the probe to capture the cuboid bone along the short axis. The cuboid height was defined as the shortest distance from the PVC plate to the cuboid (Figure 3). To reduce measurement errors, the cuboid height was calculated three times from the same image, and the mean value was used as the representative value. A second ultrasonographic measurement was performed 2 weeks later for all participants to assess reliability. During the second measurement, each examiner conducted the procedure independently to verify interrater reliability.

### 2.5 Statistical Analysis

All statistical analyses were performed using the modified R Commander 4.3.2. Pearson's correlation coefficient was used to evaluate the validity of the cuboid height measurements obtained by radiography and ultrasonography, with the significance level set at < 5%. If a significant correlation was observed, a Bland–Altman analysis was conducted to verify the absolute reliability. Systematic bias was determined by calculating the 95% confidence interval of the mean difference, and was considered present if the interval did not include zero. Proportional bias was assessed by calculating Pearson's correlation coefficient in the Bland–Altman analysis, with a significance level of < 5%, indicating the presence of proportional bias.

The intrarater reliability of cuboid height measurements taken by the same examiner (D.K.) on Days 1 and 2 and the interrater reliability between the two examiners (D.K. and Y.U.) were assessed using intraclass correlation coefficients (ICCs). ICC values were interpreted as follows: < 0.2 = slight, 0.21–0.40 = fair, 0.41–0.60 = moderate, 0.61–0.80 = substantial, and 0.81–1.00 = almost perfect [21].

## 3 Results

The mean cuboid height measured by radiography was 18.51 ± 3.97 mm. The mean cuboid heights measured by ultrasonography were as follows: 17.78 ± 3.89 mm on Day 1 by D.K., 17.90 ± 3.82 mm on Day 2 by D.K., and 17.96 ± 3.69 mm by Y.U.

### 3.1 Validity

**FIGURE 4:**
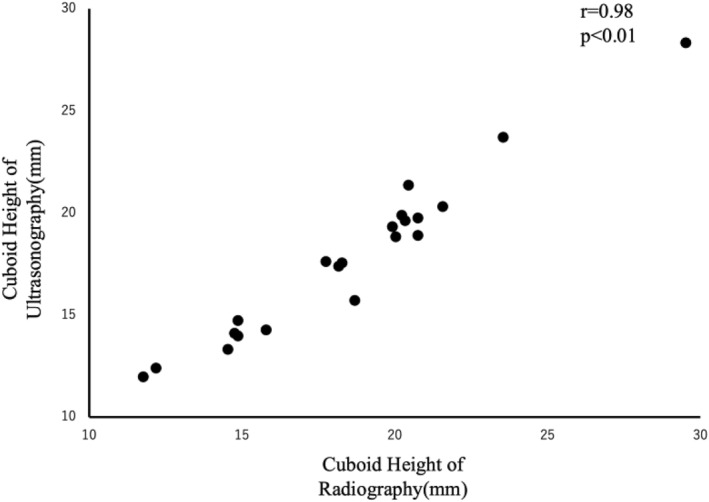
Correlation between cuboid heights measured by radiography and ultrasonography.

There was a significantly high correlation (*r* = 0.98, *p* < 0.01) between cuboid heights measured by radiography and ultrasonography (Figure 4). Bland–Altman analysis revealed a fixed bias of −0.71 mm (95% confidence interval: −0.96 to −0.46 mm) for ultrasonographic measurements compared to radiographic measurements (*p* < 0.01). No proportional bias was observed (*r* = 0.25, *p* = 0.92).

### 3.2 Reliability

Table 1 presents the ICCs for the intrarater and interrater reliability of cuboid height measured by ultrasonography, along with 95% confidence intervals and standard errors. The intrarater reliability was ICC (1.1) = 0.98, indicating almost perfect reliability. The interrater reliability was ICC (2.1) = 0.99, indicating almost perfect reliability.

**TABLE 1:** Intraclass correlation coefficients for the cuboid height measured by ultrasonography.

	ICC (1.1)	95% CI		SEM (mm)	ICC (2.1)	95% CI		SEM (mm)
		Lower	Upper			Lower	Upper	
Cuboid Height	0.98	0.97	0.99	0.52	0.99	0.97	0.99	0.41

Abbreviations: CI, confidence interval; ICC, intraclass correlation coefficients; SEM, standard error of mean.

## 4 Discussion

This study had two objectives: first, to determine the correlation between cuboid height measurements obtained using radiography and ultrasonography, and second, to evaluate the intrarater and interrater reliabilities of cuboid height measurements using ultrasonography.

First, the correlation coefficient between cuboid height measurements using radiography and ultrasonography was 0.98, indicating a strong correlation. Additionally, Bland–Altman analysis revealed a fixed bias of −0.71 mm (95% CI: −0.96 to −0.46 mm), indicating that ultrasonography consistently measured slightly smaller values compared to radiography. This discrepancy may be attributed to different measurement perspectives; radiography measures the lateral aspect of the cuboid, whereas ultrasonography measures the plantar aspect. The landmarks used in this study likely corresponded to the articular surface of the os peroneum.

On radiography, the lateral aspect of the cuboid includes a protrusion associated with the fibular groove and the path of the peroneus longus tendon [22]. This explains the depiction of the cuboid height in Figure 1. Conversely, ultrasonography likely captures the articular surface of the os peroneum from the plantar side. Anatomical studies using cadavers have reported that approximately 75% of cuboid bones in South Indians have an elliptical or circular articular surface for the os peroneum on the plantar side, with a distal fibular groove [23]. Given that the measurements in this study were taken proximomedially from the fifth metatarsal tuberosity, the identified fixed bias may have resulted from the different measurement planes. However, because the fixed bias was < 1 mm and ultrasonography results closely approximated radiographic values, ultrasonography can be considered a viable alternative for measuring cuboid height.

Measurement reliability is critical when the cuboid height is used as an outcome of therapeutic interventions. In this study, intrarater and interrater reliability were almost perfect, with ICC values of 0.98 and 0.99, respectively. This high reliability is likely attributable to the use of a PVC plate in the custom‐measuring platform. The plate ensured consistent probe pressure and angle, thus minimizing measurement errors associated with variations in probe handling.

Although ultrasonography enables real‐time observations of musculoskeletal structures, the probe angle and pressure can affect the brightness and distance measurements, leading to potential errors [18, 19]. Previous studies have reported that changes in the probe angle from 2° to 6° can affect brightness by 4.7%–10.5%, with a maximum coefficient of variation of 24.5% [18]. Additionally, excessive probe pressure can compress the plantar soft tissues, thereby affecting arch height reliability. The use of a PVC plate mitigated these factors, resulting in highly reliable measurements.

In conclusion, this study demonstrated a strong correlation between cuboid height measurements obtained using radiography and ultrasonography with high intrarater and interrater reliabilities. These findings suggest that ultrasonography is suitable for evaluating the LLA.

### 4.1 Clinical and Research Implications

Traditionally, evaluation of the cuboid and the fifth ray of the foot relies on subjective assessments and manual techniques [12, 13, 14]. The ability to quantitatively evaluate cuboid height would enable objective assessment of the lateral foot and aid in therapeutic interventions. Quantitative evaluation of the lateral foot also holds the potential for further research. For example, it could provide insights into the mechanisms underlying fifth metatarsal stress fractures, which are common in soccer players. Fifth metatarsal stress fractures have not yet been evaluated in the midfoot, despite the existence of metatarsal morphology, forefoot evaluation [15, 24], and hindfoot static evaluation of calcaneal inversion and eversion [15]. We propose to measure cuboid height as one of the metrics for the evaluation of the midfoot, which could be used as an outcome. Quantitative data can provide valuable outcomes for such studies.

### 4.2 Limitations

This study had certain limitations. First, the sample size was small, and the subject had limited physical characteristics. The participants were limited to Japanese university students, which restricted the range of foot sizes studied. Previous research using radiography reported a maximum cuboid height of 1.9 cm, lower than the values observed in this study, which reached up to 2.95 cm [11]. Although this study included participants with relatively high cuboid heights, it is unclear whether similar results would be obtained in populations with larger feet or different demographics. Furthermore, it is unclear whether similar results would be obtained with different physical characteristics, such as age, height, and weight, if the foot morphology was a pronated or supinated foot.

Second, it should be noted that the US alone cannot perform measurements, and the custom standing platform must be prepared, and it may be disadvantageous for measurements in the field.

Third, this study focused on static measurements. The use of a custom platform to position the ultrasound probe limits its application to dynamic scenarios, such as walking or other movements, making it challenging to measure the dynamic behavior of the cuboid.

## 5 Conclusion

Cuboid height measurements using ultrasonography demonstrated a strong correlation with radiographic findings and high intrarater and interrater reliability. These findings suggest that ultrasonography is a valid and reliable tool for evaluating the LLA.

## Author Contributions

**Daichi Kawamura:** conceptualization, data curation, formal analysis, funding acquisition, methodology, validation, visualization, writing–original draft, writing–review and editing. **Takashi Komatsu:** methodology, writing–review and editing. **Masanobu Suto:** data curation, writing–review and editing. **Hikaru Narita:** data curation, writing–review and editing. **Yasuyuki Umezaki:** data curation, writing–review and editing. **Saki Takahashi:** funding acquisition, writing–review and editing. **Hiroshi Shinohara:** methodology, supervision, writing–review and editing.

## Ethics Statement

This cross‐sectional study was approved by the ethics committee of the Komatsu Orthopedic Sports Clinic (Ethics Approval Number: 2024.02).

## Consent

Informed consent was obtained from all patients involved in this study.

## Conflicts of Interest

The authors declare no conflicts of interest.

## Data Availability

All relevant data are included in the main text of the article in the form of tables and figures.
